# Dynamic-Sensitive centrality of nodes in temporal networks

**DOI:** 10.1038/srep41454

**Published:** 2017-02-02

**Authors:** Da-Wen Huang, Zu-Guo Yu

**Affiliations:** 1 Key Laboratory of Intelligent Computing and Information Processing of Ministry of Education and Hunan Key Laboratory for Computation and Simulation in Science and Engineering, Xiangtan University, Xiangtan, Hunan 411105, China; 2 School of Mathematical Sciences, Queensland University of Technology, GPO Box 2434, Brisbane, Q4001, Australia

## Abstract

Locating influential nodes in temporal networks has attracted a lot of attention as data driven and diverse applications. Classic works either looked at analysing static networks or placed too much emphasis on the topological information but rarely highlighted the dynamics. In this paper, we take account the network dynamics and extend the concept of Dynamic-Sensitive centrality to temporal network. According to the empirical results on three real-world temporal networks and a theoretical temporal network for susceptible-infected-recovered (SIR) models, the temporal Dynamic-Sensitive centrality (TDC) is more accurate than both static versions and temporal versions of degree, closeness and betweenness centrality. As an application, we also use TDC to analyse the impact of time-order on spreading dynamics, we find that both topological structure and dynamics contribute the impact on the spreading influence of nodes, and the impact of time-order on spreading influence will be stronger when spreading rate b deviated from the epidemic threshold b_*c*_, especially for the temporal scale-free networks.

Centrality is a fundamental concept in network analysis, and how to measure the centrality of nodes has become an essential part of analysing and understanding networked systems including social networks^1^,^2^,^3^, biology networks^4^,^5^,^6^, Internet^7^,^8^, ecological networks^9^, traffic networks^10^. In networked system, some significant nodes are responsible for broadcasting information and therefore locating and protecting them are crucial for the whole information processing system^11^,^12^. The definition of centrality fails to reach an agreement, it leads to existing many methods to evaluate node importance. Most of conventional methods combine the definition of centrality closely with the networks topological structure^13^,^14^,^15^,^16^, these methods emphasize that the importance of nodes is related to the topological parameters, such as degree, closeness, betweenness, *k*-shell^15^, H-index^16^. Sometime these methods perform well while we are considering the structural importance of nodes, such as the path length and the number of paths. But they may not perform well in the processes like spreading which consider more about the dynamics of nodes^17^. As an extensive process, spreading can describe many important activities in the real-world, including the outbreak of epidemics^18^,^19^,^20^, the spread of news and ideas^21^,^22^, the rise of political movements^23^,^24^. The early method of describing the spreading process starts in the fields of epidemiology^18^,^25^. This years, at the core of all data-driven modeling approaches lies the structure of human interactions, mobility, and contact patterns that finds its best representation in the form of networks^26^,^27^,^28^, but the key problems that general solution of the master equation of the networked system is hardly achievable even for very simple dynamical processes^29^. The numerical simulation has been a good reference tool to find the approximate solutions of the spreading process, but when the scale of networks become large, numerical simulation may be ineffective. Recently, Morone and Makse developed the theory of influence maximization in complex networks and introduced a new method to find influencers via optimal percolation^11^, they found that some weakly connected nodes can become top influencers in large networks when they act as bridges among node clusters, the new method and its improved version make it possible to find influencers in large social media^30^.

Beside the topological features, recent studies indicate that the dynamics also play an important role in spreading process^17^,^31^,^32^,^33^. For example, if we change the spread rate in epidemic process, although the networks topological structure remain the same but the results of spreading may be totally different. There are few works taking into account the properties of the underlying spreading dynamics^17^,^34^,^35^. Klemm *et al*. suggested that the eigenvector centrality can be used in estimating dynamical influences of nodes in the susceptible-infected-recovered (SIR) spreading model^34^, and theoretical proof had been given to show that there exist a closed relation between the infection scale and eigenvector centrality in susceptible-infected-susceptible (SIS) model of the networks^35^. Liu *et al*.^17^ gave a definition of centrality (Dynamics-Sensitive centrality) of static networks while only use three parameters to describe the spreading influence, the numerical simulation shows that the new centrality performs much better than some topological structural centralities like degree, *k*-shell and also is of comparable with eigenvector centrality.

However, all above mentioned methods are restricted to static networks. In real life many networks are temporal^36^,^37^,^38^,^39^,^40^,^41^,^42^,^43^, namely the networks topological structure evolving with time, and many studies have shown that time-order have great influence not only on the topological structure but also the spreading dynamics of this kind of general networks^41^,^44^,^45^. There are many temporal versions of centrality which have been used to describe the structural importance of nodes^44^,^46^,^47^,^48^, but these methods only take into account the topological features. Since the dynamics are much more complicated in the networks with the variety of the topological structure, and more attention should be paid for this kind of general networks. It should be a significant work to investigate the dynamics on temporal networks.

In the past a few years, our group investigated the multifractal scaling properties of unweighted and weighted networks^49^,^50^,^51^,^52^. In this paper, we consider more about the spreading dynamics of nodes and extend the work of Liu *et al*.^17^ to a more general and more realistic model. According to the empirical results on three real-world temporal networks and a theoretical temporal network, the extended method performs much better than other six methods. As an application, we use it to analyse the influence of time-order in temporal networks.

## Visualization model

**Figure 1:**
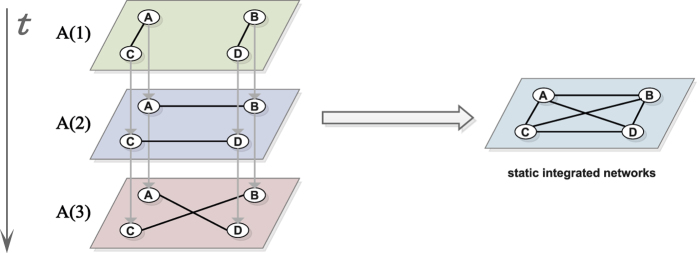
An example temporal network represented by using a multilayer network structure and its static integrated networks.

A temporal network with *N* nodes and time length *T* is defined as a sequence of *L* time snapshots of equal size *δ* = *T*/*L*, we can use multilayer network structure^53^ to represent temporal networks, where each time snapshot can be treated as networks interaction inter-layer, and all nodes naturally connect themselves between layers. Fig. 1 is an example of temporal network represented by using a multilayer network structure, there are four nodes A, B, C, D with *T* = 3, *L* = 3,*δ* = 1. If node *i* has a connection with node *j* at time snapshots *t*, where *t* ∈ [1, …, *L*], then *i* and *j* are connected by undirected line at time layer *t*. In a temporal network with *N* nodes, the topological structure at time layer *t* can be described by the adjacency matrix *A*(*t*) = {*a*(*t*)}_*ij*_. As shown in Fig. 1:


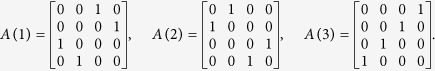


As a tool to measure the influence of nodes, dynamics-sensitive centrality^17^ performs well in static networks. But for more general dynamical networked system, the use of it has been restricted. Now we use the above visualization model and extend dynamics-sensitive centrality to more general situation.

## Temporal dynamics-sensitive centrality

We consider the susceptible-infected-recovered (SIR) model in discrete time, where individuals (i.e., the nodes of the network) can only be in one of three mutually exclusive states: susceptible (S), infectious (I), recovered (R). At each time step, a node in the state S will get infected with infection probability *β* when contacting one of its infected neighbors. And an infected node can be recovered with a probability *μ*. When we set *μ* = 0, the SIR model reduces to a standard SI model: nodes can only be susceptible or infected. We consider the temporal network forming the substrate of the spreading process to be a sequence of undirected and unweighted temporal networks, which has been described above. We used a Markov chain for the epidemic model and derive the analytical result of node influence. Denote **x(t)**(*t* ≥ 0) as an *n* × 1 vector whose components are the probabilities of nodes to be ever infected no later then the time step *t*, then **P**(**t**) = **x(t)** − **x**(**t−1**)(*t* ≥ 1) is the probabilities of nodes to be infected at time step *t*, and we denote **P**(**0**) = **x**(**0**) to represent the initial condition. If *i* is the only initially infected node, then *x*_*i*_(0) = 1. Notice that, **x(t)** is the cumulative probability that can be large than 1, and we use the term probability just for simplicity. In the first time step, **x**(**1**) = *βA*(1)**x**(**0**), and in the Methods Section, we prove that for *t* > 1 the following equation can be obtained:


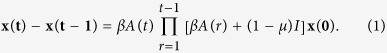


So we have


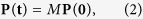


where 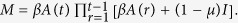


The probabilities of nodes to be ever infected no later than the time step *t* in temporal networks can be expressed by


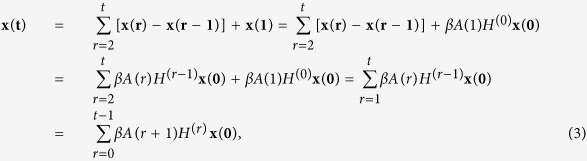


where 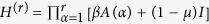, and we denote *H*^(0)^ = 1. Aral and Walker^54^ showed that the attributes of nodes are highly correlated with influence of nodes and tendencies to be influenced, which indicates that we can use the sum of infected probabilities of all nodes to express the the spreading influence of nodes. Denote *S*_*i*_(*t*) to be the sum of infected probabilities of node *i* at time step *t*, then


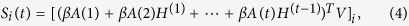


where *X*^*T*^ means the transpose of matrix *X, V* = (1, 1, …. 1)^*T*^ is *n* × 1 vector whose components are all equal to 1. Obviously, *A*(*t*)^*T*^ = *A*(*t*), so


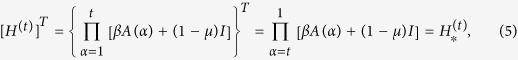


then the spreading influence of all nodes can be described by the vector


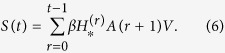


If we let *A* = *A*(1) = *A*(2) = … = *A*(*t*), then the temporal networks are static. In this particular case, 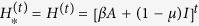 is a symmetric matrix, then 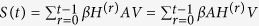, which is in accordance with the result obtained by Liu *et al*.^17^.

Now we have established the method to measure the spreading influence of nodes in temporal networks. But there are three assumptions need to be explained. First, we follow Refs 17 and 55 and use a linear coupling instead of nonlinear coupling to calculate the probabilities of the nodes to be infected at time step *t*, namely, if a susceptible node has *m* infected neighbors, then the probability of the node to be infected is approximated as *mβ*, instead of 1 − (1 − *β*)^*m*^. Second, the cumulative probability **x(t)** can be larger than 1, which is not mathematically rigorous. As infected probability of every node is overestimated, here we take the cumulative probability **x(t)** just as a measure of influence. And we assume that **x(t)** is meaningful and has a closed relation with the spreading influence. The performance of the operation need to be tested in real networks, and this will be extended in the following section. Third, we assume periodic boundary conditions for the network dynamics, here we regard *L* as the total number of network time snapshots. i.e. *A*(*L* + 1) = *A*(1), where *L* is arbitrary and finite, this processing method causes no loss in generality and has been used by Valdano *et al*.^56^. With different recovery rate *μ*, the time-varying structure of temporal networks is able to alter the value of the epidemic threshold *β*_*c*_. We think the assumption is meaningful because when *L* → ∞ and *β* > *β*_*c*_, all nodes in the networks would be infected no matter which node is the source of the disease, in this special condition we could not distinguish the spreading influence between nodes. For a given recovery rate *μ*, Valdano *et al*.^56^ also tested that it would affect the epidemic threshold *β*_*c*_ estimation only for rather small values of *L*, the asymptotic solutions of epidemic process are periodic of period *L*. From Eq. (2) and the same processing method^56^, we have the spectral radius of *M* meets:


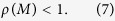


Eq. (7) makes sure that 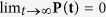, this yields the threshold condition *ρ*(*M*) = 1, then the epidemic threshold *β*_*c*_ can be calculated^56^,^57^.

## Results

### Evaluating the spreading performance

**Figure 2:**
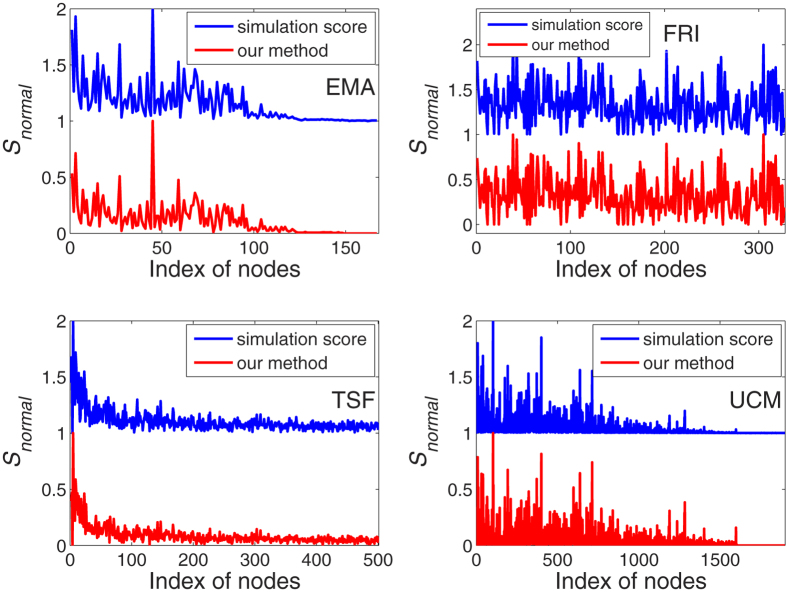
The infection probability *β* = 0.01, and the recovered probability *μ* = 0.1, the time step of the four networks are set by *t* = 10. Numerical simulation point is obtained by averaging over 10^4^ independent runs, and the Kendall’s Tau coefficients between the results of the method and the standard SIR model are 0.9265, 0.9443, 0.7866, 0.8514 respectively of the EMA, FRI, TSF, UCM.

In the following experimental results, we demonstrate the effectiveness of the method by comparing with the SIR model of varying spreading rate *β*. Three real-world temporal networks, including *Email networks* (EMA)^58^, *High school friendship relations networks* (FRI)^59^, *University of California messages networks* (UCM)^60^, and a theoretical temporal scale-free network (TSF) generated by Barabasi-Albert (BA) model^61^, are used in our empirical analysis, the data description can be found in Methods section. We calculated the spreading influence by both our method and SIR spreading model (see Methods section for the description of SIR spreading model). As shown in Fig. 2, we perform the method with *β* = 0.01, *μ* = 0.1, and the time step is set by *t* = 10 (*t* ≤ *L, L* has been given in Table 1), and a normalization can be performed without a loss of precision:


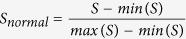


In order to compare between the results of the method and the results of SIR simulation but without a loss of precision, the simulation results use translation transform of *S*′ = *normal*(*S*) + 1 after the normalization. the Kendall’s Tau coefficients^62^ between the results of the method and the standard SIR model are 0.9265, 0.9443, 0.7866, 0.8514 respectively of the EMA, FRI, TSF, UCM which show a high effectiveness of the method.

**Figure 3:**
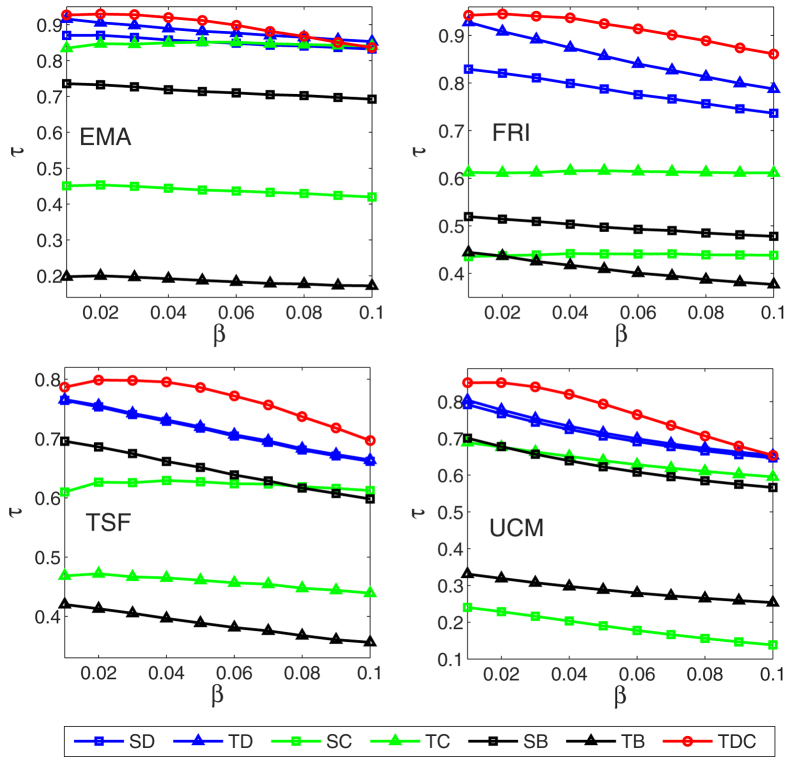
The accuracy of seven centrality measures in evaluating the spreading influence of nodes according to the SIR model in the four networks, quantified by the Kendall’s Tau coefficients. The spreading rate *β* varies from 0.01 to 0.10, recovery rate *μ* = 0.1, and the time step is set as *t* = 10. Each data point is obtained by averaging over 10^4^ independent runs.

In addition, three well-known centrality measure methods *degree centrality, closeness centrality, betweenness centrality*, and their temporal version methods introduced by Kim *et al*.^48^ are considered, the details of these methods can be seen in the Methods section. We applied these six methods to the four temporal networks, both the results of above six methods and ours are compared with the simulation results of the standard SIR model. In the standard SIR model, the spreading rate *β* varies from 0.01 to 0.10 with a step of 0.01, the recovery rate *μ* is set by 0.1, and the time step is set as *t* = 10. As shown in Fig. 3, each data point is obtained by averaging over 10^4^ independent runs, here SD, TD, SC, TC, SB, TB, TDC, SIR represent static degree, temporal degree, static closeness, temporal closeness, static betweenness, temporal betweenness, temporal dynamics-sensitive centrality, and simulation results of SIR model respectively. Temporal dynamics-sensitive centrality (TDC) proposed in this paper obtains much higher accuracy than other six methods through the Kendall’s Tau coefficient *τ*.

Moreover, we compare the consumed CPU time of the above eight methods in Table 2. All experiments are tested on a computer: Intel Core i3 2.53 G Hz with RAM DDR3 4 GB and 1.6 GHz. From Table 2, we can see that local centrality methods like static degree (SD) and temporal degree (TD) consumed least time, temporal dynamics-sensitive centrality (TDC) also performed well, and global centrality methods like static closeness (SC), temporal closeness (TC), static betweenness (SB), temporal betweenness (TB) consumed a substantial amount of computation time and memory resources. Although numerical simulation of SIR did not take a substantial amount of memory resources, it consumed too much time and sometime it was unbearable.

### Dynamics of spreading analysis on null models

In the study of statistical properties of networks, it’s not enough to assess the importance, unexpectedness, or underrepresentation of topological features of empirical networks only by calculating the value of some topological statistics, a common way to deal with the problem is by comparing the features against some reference model where the network is randomized, and these randomized network models are called null models. More about null models, readers can refer to Refs 41 and 45.

Whether or not taking into consideration of the influence of time is the difference between statics networks and temporal networks. In temporal networks, time-order of the event sequences between nodes not only have influence on the networks topology structure, but also the spreading dynamics^41^,^44^,^45^. Tang *et al*.^44^ pointed out that a path is not reachable in temporal networks, but may be reachable when turning temporal networks into static networks. Karsai *et al*.^45^ showed that randomizing the time-order will slow down the spreading. In this paper, we care more about the effects of time-order on the spreading influence of nodes, we apply the above analysis to null models where the time-order of original event sequences are randomized. Algorithmically, the method of the model is defined as follows:

1. **Step 1** Go over all edges sequentially.
2. **Step 2** For every edge 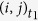 at time *t*_1_, randomly pick another time *t*_2_.
3. **Step 3** Replace the edge 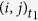 and 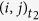, namely only change the time-order of the events happen between the same two nodes.
4. **Step 4** Repeat the above processes until the time orders are completely randomized.

**Figure 4:**
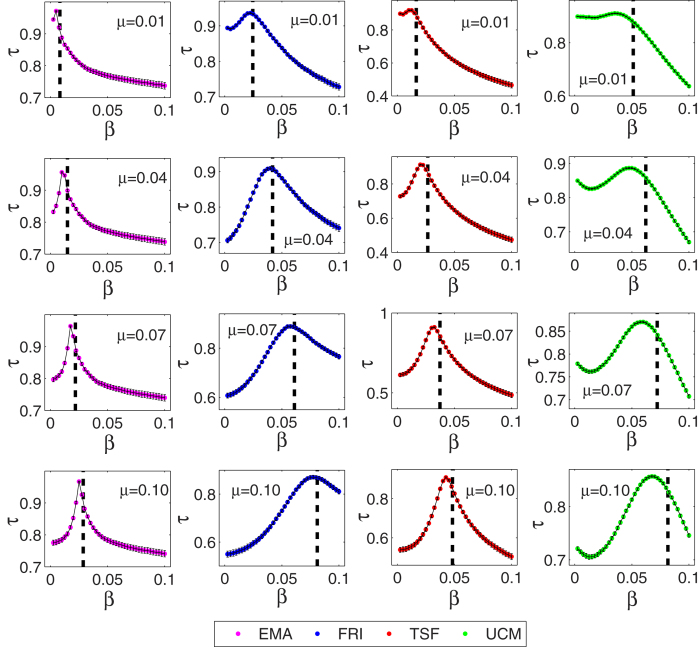
Kendall’s Tau coefficients between original networks and randomly reset networks. Here, black dotted line represent the epidemic threshold *β*_*c*_. The recovery rate *μ* was set by 0.01, 0.04, 0.07, 0.10, for each recovery rate, the spreading rate *β* varies from 0.0025 to 0.10 with a step of 0.0025, and the time step is set as *t* = *L*. Each data point is obtained by averaging over 10^3^ independent runs. Each error bar covers two standard deviations for all the results.

Denoted *E*(*i, j*)_*t*_ = 1 if *i* and *j* is connected at time *t*, or *E*(*i, j*)_*t*_ = 0 means disconnected otherwise. It is easy to find that if 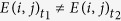, then above randomly replace process between time *t*_1_ and *t*_2_ will be valid. This random method ensemble conserves the set of times of the original contact sequence, but destroys burstiness of events on individual vertices and edges as well as correlations between events such as triggered chains, the aggregated rate of events in the network is unchanged and will still follow the typical circadian and weekly patterns of human activity^41^,^45^. In the experiment, the temporal length of the four networks were chosen by *t* = *L*, we apply the null model to the spreading process while the recovery rate *μ* is set by 0.01, 0.04, 0.07, 0.10, and infection rate *β* is set by a range from 0.0025 to 0.1 with a step of 0.0025 respectively for each recovery rate *μ*. For every pair of (*μ*,*β*), the original networks are randomly reset 1000 times by using the null model algorithm, denoted *S*′(*t*) the mean of spreading influence of 1000 random networks, the Kendall’s Tau coefficients between the *S*(*t*) of the original networks and *S*′(*t*) are used to measure the the impact of the time-order, and larger the value of Kendall’s Tau coefficients, the impact of time-order is small. The epidemic threshold *β*_*c*_ respectively for the four networks are shown in Table 3. As shown in Fig. 4, we are surprised to find that the impact of time-order on spreading influence will be stronger when *β* deviated from the epidemic threshold *β*_*c*_, especially for the temporal scale-free networks. When *β* is set by the epidemic threshold condition *β*_*c*_ of the four networks, the Kendall’s Tau coefficients between *S*(*t*) of the original networks and *S*′(*t*) of the random networks can be larger than 0.84, which shows a small impact of time-order on spreading influence. The detailed results of Kendall’s Tau coefficients can be found in Supplementary Information.

## Discussion

Centrality is a important topic with many diverse applications, and how to measure the centrality of nodes has become an essential part of analysing and understanding networked systems. As summarized in papers^14^,^63^,^64^, most of previous work concentrate on the topological information but rarely highlight the dynamics^17^. Liu *et al*.^17^ showed that dynamics-sensitive centrality in static networks performed much better than some topological structural centrality in spreading process, this inspire us to consider the problem in a more general dynamics networks, where networks topological structure evolving with time. In this paper, in addition to the topological information, we take the time into consideration and propose temporal centrality metrics based on the time-ordered graph. According to the empirical results on three real-world temporal networks and a theoretical temporal network, the extended method (TDC) perform much better than other six methods in the early stage of spreading. Although it is unfair to directly compare TDC with other six methods because TDC takes account more parameters, the method enables us to investigate the dynamics in spreading process while only use three parameters, we can use it to detect the potential super-spreaders for epidemic control in temporal networks. The method in this paper has advantage over the method proposed by Liu *et al*.^17^, it not only can be applied to static networks, but also can be used in more general networks.

The dynamics are much more complicated in temporal networks for the variety of the topological structure. To the best of our knowledge, there are few works had ever investigated the impact of time-order on the spreading influence of nodes. As an application, TDC enable us to analyse the impact of time-order on spreading dynamics, we are surprised to find that both topological structure and dynamics contribute the impact on the spreading influence of nodes, but the impact of time-order on spreading influence will be stronger when *β* deviated from the epidemic threshold *β*_*c*_. A direct application of this conclusion can be that when dynamics parameter *β* approach epidemic threshold *β*_*c*_, we can still find the super-spreaders for epidemic only rely on the static integrated networks and the number of each edge links. We think our method can be extend to more dynamics analyses such as the impact of edge sequences on spreading dynamics. All in all, there are many problem remain to be solved since we conclude that both topological structure and dynamics contribute the impact on the spreading influence of nodes, but the specific mechanisms involved remain to be explored.

## Methods

### Derivation of Eq. (1)


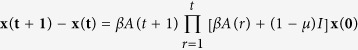


Proof: In the first time step, **x**(**1**) = *βA*(1)**x**(**0**), then when *t* = 1,


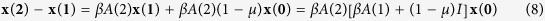


We assume that when *t* ≤ *p*, the following equation can be established


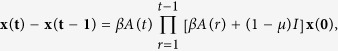


then for *t* = *p* + 1, we have


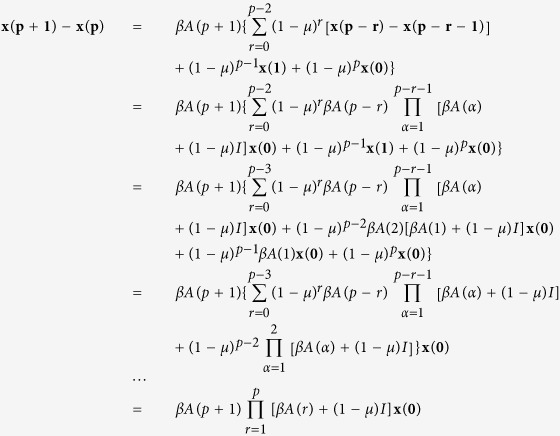


which completes the proof.

### Benchmark methods

We consider two kinds of benchmark methods here, one type are static networks methods which have been widely used over last years, such as *degree centrality, closeness centrality*, and *betweenness centrality*. The other are temporal networks methods which have been developed these years, the representative are temporal type of degree centrality, closeness centrality, and betweenness centrality.

The *degree centrality* of node *i* is defined as the number of links incident upon it, namely


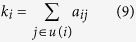


where *a*_*ij*_ is the element of matrix *A. u*(*i*) is the neighbor nodes of node *i*. Degree centrality is a local centrality, and works especially well in evaluating the spreading influence of nodes when the spreading rate *β* is small.

*Closeness centrality*^65^ of node *i* is defined by the reciprocal of sum of its distances from all other nodes.


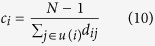


where *N* is the total number of nodes, *d*_*ij*_ is the shortest path length between node *i* and node *j*. Closeness centrality is a global centrality and performs better in evaluating nodes’ spreading inflences when the spreading rate *β* is high.

*Betweenness centrality*^65^ of node *i* is defined by the number of shortest paths from all vertices to all others that pass through node *i*.


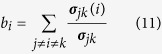


where *σ*_*jk*_ is the total number of shortest paths from node *j* to node *k*, and *σ*_*jk*_(*i*) is the number of those paths that pass through node *i*. Betweenness centrality is also a global centrality and has been attached with wide application, including computer and social networks, biology, transport and scientific cooperation.

Tang *et al*.^44^ proposed a method to identify important nodes using temporal versions of conventional centrality metrics, Kim *et al*.^48^ extend Tang’s work to a more general and more realistic model. Readers can refer to Tang *et al*.^44^ or Kim *et al*.^48^ for the details of the temporal versions of conventional centrality. In this paper, we applied the above three conventional centrality metrics and the temporal version of these three centrality metrics proposed by Kim *et al*.^48^.

In fact the static networks can be regarded as a integrated process of temporal networks regardless of the influence of time-order^42^, and conventional centrality methods are no more suitable for this kind of general networks, in order to solve the problem and compare the performance of the conventional static methods with our methods, we first convert the temporal networks into static integrated networks (see Fig. 1), and then calculating the centrality by using the three conventional centrality methods. We also calculate the temporal versions of conventional centrality by using Kim’s methods, both of these results are considered as benchmark to be compared with the results of our method, and the results are shown in Results section.

### SIR Spreading Model

Initially, we set one node to be in the initial infected state I, this node corresponds to our single spreader (in general, the initial node can be any node of the graph, here in the simulation we run over all nodes of networks; the same procedure is also performed for the baseline methods). The rest of the nodes are assigned to the susceptible state S. At each time step, the infected nodes can infect their susceptible neighbors with probability *β* (infection rate). Furthermore, the nodes that have been previously infected can recover from the disease with probability *μ* (recovery rate). The process is repeated within the given time step *t*(*t* ≤ *L*). We calculate the number of infected nodes *N*_*i*_(*t*) after *t* steps the disease firstly spreads from the initial node *i*, and use *N*_*i*_(*t*) to represent the simulation spreading influence of node *i*.

### Data Sets

In order to demonstrate the effectiveness of the method, we performed the numerical analysis mainly using the following empirical networks.

- *Email networks*(EMA)^58^. This network represented the history of internal e-mail communication between employees of a small-sized manufacturing.
- *High school friendship relations networks*(FRI)^59^. This network was a medium-sized data set correspond to the contacts and friendship relations between students in a high school.
- *Temporal scale-free networks*(TSF). This network was a combination of 100 snapshots, and each snapshots was generated by Barabasi-Albert *BA* model^61^.
- *University of California messages networks*(UCM)^60^. This network contains sent messages between the users of an online community of students from the University of California, Irvine.

In Table 1, we provide some detailed statistical properties of the above networks. For our analyses, if not stated otherwise, these networks are both undirected and unweighted networks. EMA, FRI, UCM are real-world networks which represent human interactions in diverse social contexts and have different topological and temporal characteristics, TSF is a theoretical network generated by *BA* scale-free model.

## Additional Information

**How to cite this article**: Huang, D.-W. and Yu, Z.-G. Dynamic-Sensitive centrality of nodes in temporal networks. *Sci. Rep.* **7**, 41454; doi: 10.1038/srep41454 (2017).

**Publisher's note:** Springer Nature remains neutral with regard to jurisdictional claims in published maps and institutional affiliations.

## Supplementary Material

Supplementary Information

## Figures and Tables

**Table 1:** Statistical properties of the networks used in our analyses, where *N, E, L, δ* denoted by the number of nodes, the number of links, temporal length, and maximum snapshots size respectively for the networks.

Network	*N*	*E*	*L*	*δ*
EMA	167	82927	272	1day
FRI	327	188508	101	1hour
TSF	500	36684	100	N/A
UCM	1899	59835	39	per 5 days

**Table 2:** The CPU time of the eight algorithms for the four networks (unit: s).

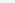	EMA	FRI	TSF	UCM
SD	0.009	0.01	0.022	0.2
TD	0.005	0.007	0.018	0.19
SC	0.07	0.49	2.24	255
TC	3.0	10.7	29.2	6097
SB	0.024	0.11	0.26	2.81
TB	6.3	22.9	69.9	1275
TDC	1.0	0.6	1.3	34
SIR	1113	2762	5039	42302

**Table 3:** The epidemic threshold *β*_*c*_ respectively for the four networks.

Network	*μ* = 0.01	*μ* = 0.04	*μ* = 0.07	*μ* = 0.10
EMA	0.008327	0.01494	0.02179	0.02878
FRI	0.024551	0.04190	0.06100	0.08093
TSF	0.016572	0.02664	0.03726	0.04824
UCM	0.051286	0.06223	0.07220	0.08152
