# Supplementary material for: Dynamic-Sensitive centrality of nodes in temporal networks
Source: Sci Rep. 2017 Feb 2;7:41454. doi: 10.1038/srep41454 (PMC5288707; doi:10.1038/srep41454)
Supplement: Supplementary Information [file srep41454-s1.doc]

**Dynamic-Sensitive centrality of nodes in temporal networks**

**Da-Wen Huang^1^ and Zu-Guo Yu^1,2,*^**

^1^Key Laboratory of Intelligent Computing and Information Processing of Ministry of Education and Hunan Key Laboratory for Computation and Simulation in Science and Engineering, Xiangtan University, Xiangtan, Hunan 411105, China.

^2^School of Mathematical Sciences, Queensland University of Technology, GPO Box 2434, Brisbane, Q4001, Australia.

*Corresponding author: yuzuguo@aliyun.com

**Supplementary Information:** We applied the null model to the spreading process while the recovery rate$\mu$was set by 0.01, 0.04, 0.07, 0.10 (as shown in Table 1~4), and infection rate$\beta$was set by a range from 0.0025 to 0.1 with a step of 0.0025 respectively for each recovery rate$\mu$. For every pair of ($\mu$,$\beta$), the original networks were randomly reset 1000 times by using the null model algorithm, Denoted ***S’(t)*** the mean of spreading influence of 1000 random networks, the Kendall’s Tau coefficients between the ***S(t)*** of the original networks and ***S’(t)*** are shown in Table 1~4. The maximum of each column has been in bold.

**Table 1S**. $\mu$=0.01, the Kendall’s Tau coefficients between the ***S(t)*** of the four networks and ***S’(t)***.

| β | EMA | FRI | TSF | UCM |
| --- | --- | --- | --- | --- |
| 0.0025 | 0.94501 | 0.894431 | 0.898522 | 0.898914 |
| 0.005 | **0.971518** | 0.891121 | 0.894574 | 0.897395 |
| 0.0075 | 0.920481 | 0.894903 | 0.905303 | 0.897643 |
| 0.01 | 0.888056 | 0.901618 | 0.918571 | 0.895868 |
| 0.0125 | 0.865772 | 0.909931 | **0.919276** | 0.896352 |
| 0.015 | 0.854242 | 0.919459 | 0.901964 | 0.894445 |
| 0.0175 | 0.840325 | 0.928865 | 0.873676 | 0.894366 |
| 0.02 | 0.828329 | 0.935082 | 0.843617 | 0.895197 |
| 0.0225 | 0.818701 | **0.936319** | 0.813621 | 0.898451 |
| 0.025 | 0.809943 | 0.932938 | 0.786192 | 0.900689 |
| 0.0275 | 0.8023 | 0.926069 | 0.762018 | 0.90388 |
| 0.03 | 0.795736 | 0.916946 | 0.739633 | 0.906964 |
| 0.0325 | 0.789899 | 0.907383 | 0.718488 | 0.909151 |
| 0.035 | 0.785892 | 0.897155 | 0.700206 | **0.910502** |
| 0.0375 | 0.782334 | 0.885772 | 0.68286 | 0.910012 |
| 0.04 | 0.779965 | 0.875227 | 0.667373 | 0.908083 |
| 0.0425 | 0.776275 | 0.865192 | 0.651606 | 0.904311 |
| 0.045 | 0.773524 | 0.856015 | 0.63754 | 0.898914 |
| 0.0475 | 0.771047 | 0.846429 | 0.624563 | 0.891855 |
| 0.05 | 0.768501 | 0.837527 | 0.612793 | 0.883423 |
| 0.0525 | 0.766703 | 0.828934 | 0.601736 | 0.873945 |
| 0.055 | 0.764273 | 0.819453 | 0.590818 | 0.863387 |
| 0.0575 | 0.762899 | 0.811833 | 0.580287 | 0.851794 |
| 0.06 | 0.760635 | 0.804619 | 0.571155 | 0.839538 |
| 0.0625 | 0.759258 | 0.79835 | 0.562033 | 0.827155 |
| 0.065 | 0.757412 | 0.79106 | 0.554371 | 0.813509 |
| 0.0675 | 0.755871 | 0.784958 | 0.545474 | 0.801081 |
| 0.07 | 0.754295 | 0.779121 | 0.53885 | 0.787682 |
| 0.0725 | 0.752653 | 0.774861 | 0.529994 | 0.773371 |
| 0.075 | 0.750919 | 0.769081 | 0.523111 | 0.76011 |
| 0.0775 | 0.749636 | 0.763466 | 0.516106 | 0.746006 |
| 0.08 | 0.747834 | 0.759103 | 0.50939 | 0.732769 |
| 0.0825 | 0.747098 | 0.754291 | 0.503326 | 0.719897 |
| 0.085 | 0.746179 | 0.751087 | 0.497049 | 0.707296 |
| 0.0875 | 0.744792 | 0.746517 | 0.491461 | 0.693957 |
| 0.09 | 0.742919 | 0.741745 | 0.486152 | 0.682921 |
| 0.0925 | 0.741198 | 0.738037 | 0.480402 | 0.670314 |
| 0.095 | 0.74124 | 0.733717 | 0.474776 | 0.659124 |
| 0.0975 | 0.739316 | 0.73156 | 0.470656 | 0.648026 |
| 0.1 | 0.737387 | 0.727955 | 0.465207 | 0.636878 |

**Table 2S**. $\mu$=0.04, the Kendall’s Tau coefficients between the ***S(t)*** of the four networks and ***S’(t)***.

| β | EMA | FRI | TSF | UCM |
| --- | --- | --- | --- | --- |
| 0.0025 | 0.83224 | 0.706375 | 0.727489 | 0.849388 |
| 0.005 | 0.851168 | 0.712955 | 0.735408 | 0.840394 |
| 0.0075 | 0.89071 | 0.723079 | 0.751318 | 0.833452 |
| 0.01 | **0.956222** | 0.733711 | 0.77681 | 0.828898 |
| 0.0125 | 0.946423 | 0.747439 | 0.810409 | 0.825831 |
| 0.015 | 0.899116 | 0.763885 | 0.849078 | 0.825704 |
| 0.0175 | 0.871705 | 0.782441 | 0.88719 | 0.82717 |
| 0.02 | 0.854102 | 0.801634 | **0.912604** | 0.829603 |
| 0.0225 | 0.841178 | 0.821681 | 0.910041 | 0.834556 |
| 0.025 | 0.825955 | 0.84099 | 0.884604 | 0.838724 |
| 0.0275 | 0.817559 | 0.85896 | 0.85245 | 0.844719 |
| 0.03 | 0.807607 | 0.877567 | 0.819936 | 0.851408 |
| 0.0325 | 0.798778 | 0.892429 | 0.789498 | 0.858129 |
| 0.035 | 0.791832 | 0.901925 | 0.763688 | 0.865159 |
| 0.0375 | 0.787423 | 0.907811 | 0.739233 | 0.871685 |
| 0.04 | 0.783777 | **0.909464** | 0.717639 | 0.877738 |
| 0.0425 | 0.780617 | 0.906765 | 0.6969 | 0.882638 |
| 0.045 | 0.776831 | 0.899975 | 0.678892 | 0.88546 |
| 0.0475 | 0.773805 | 0.890171 | 0.662764 | **0.886486** |
| 0.05 | 0.771445 | 0.881183 | 0.647418 | 0.886312 |
| 0.0525 | 0.769751 | 0.872153 | 0.632757 | 0.883991 |
| 0.055 | 0.767185 | 0.861435 | 0.619067 | 0.879563 |
| 0.0575 | 0.765124 | 0.851705 | 0.606569 | 0.873609 |
| 0.06 | 0.763325 | 0.842594 | 0.595601 | 0.866259 |
| 0.0625 | 0.760675 | 0.83349 | 0.584288 | 0.857479 |
| 0.065 | 0.759089 | 0.824436 | 0.574831 | 0.846773 |
| 0.0675 | 0.757952 | 0.815412 | 0.564828 | 0.836068 |
| 0.07 | 0.755297 | 0.80656 | 0.556696 | 0.824516 |
| 0.0725 | 0.754632 | 0.800069 | 0.546815 | 0.811326 |
| 0.075 | 0.752432 | 0.792733 | 0.539294 | 0.798824 |
| 0.0775 | 0.751119 | 0.786989 | 0.531278 | 0.785469 |
| 0.08 | 0.74913 | 0.781194 | 0.523183 | 0.772254 |
| 0.0825 | 0.748359 | 0.775263 | 0.516386 | 0.759172 |
| 0.085 | 0.746856 | 0.768972 | 0.509426 | 0.745814 |
| 0.0875 | 0.745515 | 0.763213 | 0.502905 | 0.73202 |
| 0.09 | 0.744181 | 0.758207 | 0.497015 | 0.719967 |
| 0.0925 | 0.742339 | 0.753743 | 0.491004 | 0.706446 |
| 0.095 | 0.741721 | 0.749194 | 0.484329 | 0.694368 |
| 0.0975 | 0.740231 | 0.745046 | 0.479532 | 0.682436 |
| 0.1 | 0.738431 | 0.740952 | 0.473798 | 0.67016 |

**Table 3S**. $\mu$=0.07, the Kendall’s Tau coefficients between the ***S(t)*** of the four networks and ***S’(t)***.

| β | EMA | FRI | TSF | UCM |
| --- | --- | --- | --- | --- |
| 0.0025 | 0.796672 | 0.607325 | 0.611758 | 0.778736 |
| 0.005 | 0.802134 | 0.610765 | 0.616244 | 0.772094 |
| 0.0075 | 0.809638 | 0.61556 | 0.622727 | 0.766659 |
| 0.01 | 0.821859 | 0.621819 | 0.636233 | 0.763455 |
| 0.0125 | 0.848542 | 0.630003 | 0.652614 | 0.761123 |
| 0.015 | 0.895036 | 0.638768 | 0.675082 | 0.761528 |
| 0.0175 | **0.96377** | 0.648608 | 0.705191 | 0.763316 |
| 0.02 | 0.932317 | 0.662677 | 0.741689 | 0.766176 |
| 0.0225 | 0.88782 | 0.675612 | 0.783745 | 0.771446 |
| 0.025 | 0.864309 | 0.691207 | 0.829869 | 0.776244 |
| 0.0275 | 0.845775 | 0.70639 | 0.875025 | 0.782629 |
| 0.03 | 0.828875 | 0.724788 | 0.906781 | 0.790441 |
| 0.0325 | 0.818834 | 0.744259 | **0.910023** | 0.798558 |
| 0.035 | 0.807744 | 0.765296 | 0.885721 | 0.807309 |
| 0.0375 | 0.800124 | 0.783814 | 0.85149 | 0.816963 |
| 0.04 | 0.791725 | 0.80407 | 0.816865 | 0.826596 |
| 0.0425 | 0.786296 | 0.822378 | 0.784755 | 0.836353 |
| 0.045 | 0.783625 | 0.840117 | 0.756164 | 0.845267 |
| 0.0475 | 0.779303 | 0.855706 | 0.73095 | 0.852532 |
| 0.05 | 0.775834 | 0.869037 | 0.708412 | 0.85996 |
| 0.0525 | 0.77366 | 0.880182 | 0.686946 | 0.865602 |
| 0.055 | 0.770988 | 0.888249 | 0.669001 | 0.86843 |
| 0.0575 | 0.768206 | **0.888518** | 0.651117 | 0.870055 |
| 0.06 | 0.766635 | 0.886961 | 0.635509 | **0.870258** |
| 0.0625 | 0.764204 | 0.881626 | 0.620607 | 0.867974 |
| 0.065 | 0.761731 | 0.87452 | 0.607539 | 0.863725 |
| 0.0675 | 0.759519 | 0.867011 | 0.595211 | 0.858262 |
| 0.07 | 0.758 | 0.859068 | 0.584556 | 0.850933 |
| 0.0725 | 0.755983 | 0.849859 | 0.572726 | 0.841475 |
| 0.075 | 0.754339 | 0.839272 | 0.563114 | 0.831594 |
| 0.0775 | 0.752451 | 0.828883 | 0.553246 | 0.820526 |
| 0.08 | 0.751138 | 0.819875 | 0.544283 | 0.808922 |
| 0.0825 | 0.750112 | 0.812428 | 0.536158 | 0.797019 |
| 0.085 | 0.74854 | 0.804233 | 0.527672 | 0.784416 |
| 0.0875 | 0.746823 | 0.79769 | 0.51973 | 0.771076 |
| 0.09 | 0.745726 | 0.790043 | 0.512608 | 0.758986 |
| 0.0925 | 0.744049 | 0.783432 | 0.505407 | 0.745228 |
| 0.095 | 0.742945 | 0.777561 | 0.498347 | 0.732371 |
| 0.0975 | 0.740979 | 0.771311 | 0.492717 | 0.720138 |
| 0.1 | 0.739819 | 0.765539 | 0.485864 | 0.707103 |

**Table 4S**. $\mu$=0.10, the Kendall’s Tau coefficients between the ***S(t)*** of the four networks and ***S’(t)***.

|  | EMA | FRI | TSF | UCM |
| --- | --- | --- | --- | --- |
| 0.0025 | 0.775402 | 0.549499 | 0.538156 | 0.721039 |
| 0.005 | 0.776731 | 0.551337 | 0.540585 | 0.715108 |
| 0.0075 | 0.780469 | 0.554172 | 0.542732 | 0.710504 |
| 0.01 | 0.784696 | 0.557705 | 0.549501 | 0.707481 |
| 0.0125 | 0.79306 | 0.562548 | 0.555582 | 0.705241 |
| 0.015 | 0.803274 | 0.566651 | 0.565077 | 0.70576 |
| 0.0175 | 0.823591 | 0.571548 | 0.578428 | 0.707182 |
| 0.02 | 0.852906 | 0.579191 | 0.595693 | 0.710098 |
| 0.0225 | 0.900761 | 0.586006 | 0.617091 | 0.714851 |
| 0.025 | **0.967024** | 0.594854 | 0.642541 | 0.719107 |
| 0.0275 | 0.925536 | 0.602774 | 0.673849 | 0.724855 |
| 0.03 | 0.881951 | 0.612733 | 0.711164 | 0.732432 |
| 0.0325 | 0.857829 | 0.624057 | 0.753839 | 0.740438 |
| 0.035 | 0.837337 | 0.637313 | 0.801074 | 0.749349 |
| 0.0375 | 0.822609 | 0.650008 | 0.850275 | 0.759774 |
| 0.04 | 0.811229 | 0.66386 | 0.890134 | 0.770199 |
| 0.0425 | 0.801689 | 0.680311 | **0.908226** | 0.780903 |
| 0.045 | 0.794042 | 0.697109 | 0.894406 | 0.792034 |
| 0.0475 | 0.787927 | 0.714052 | 0.862322 | 0.801677 |
| 0.05 | 0.783846 | 0.730369 | 0.825972 | 0.813181 |
| 0.0525 | 0.779956 | 0.748721 | 0.790888 | 0.823594 |
| 0.055 | 0.776206 | 0.766678 | 0.760156 | 0.832764 |
| 0.0575 | 0.773687 | 0.78552 | 0.731896 | 0.840883 |
| 0.06 | 0.770463 | 0.802029 | 0.707381 | 0.847998 |
| 0.0625 | 0.768144 | 0.818362 | 0.684749 | 0.85246 |
| 0.065 | 0.765377 | 0.831782 | 0.665381 | 0.855574 |
| 0.0675 | 0.762674 | 0.845594 | 0.646774 | **0.856765** |
| 0.07 | 0.760784 | 0.857426 | 0.630609 | 0.856225 |
| 0.0725 | 0.758926 | 0.864931 | 0.614763 | 0.853401 |
| 0.075 | 0.756792 | 0.869536 | 0.60092 | 0.849221 |
| 0.0775 | 0.754621 | **0.870644** | 0.58768 | 0.843454 |
| 0.08 | 0.753114 | 0.869274 | 0.576055 | 0.835518 |
| 0.0825 | 0.751728 | 0.866443 | 0.565468 | 0.827015 |
| 0.085 | 0.75079 | 0.861095 | 0.555004 | 0.817274 |
| 0.0875 | 0.748982 | 0.853897 | 0.545723 | 0.805998 |
| 0.09 | 0.747093 | 0.845283 | 0.536495 | 0.795066 |
| 0.0925 | 0.745477 | 0.835944 | 0.527886 | 0.782827 |
| 0.095 | 0.74489 | 0.827781 | 0.518543 | 0.770676 |
| 0.0975 | 0.742517 | 0.818133 | 0.511508 | 0.75872 |
| 0.1 | 0.741078 | 0.810416 | 0.50381 | 0.745712 |
